# Common types of tuberculosis and co-infection with HIV at private health institutions in Ethiopia: a cross sectional study

**DOI:** 10.1186/1471-2458-14-319

**Published:** 2014-04-07

**Authors:** Getahun Asres Alemie, Feseha Gebreselassie

**Affiliations:** 1 Department of Epidemiology and Biostatistics, College of Medicine and Health Sciences, The University of Gondar, Gondar, Ethiopia; 2 Department of Internal Medicine, College of Medicine and Health Sciences, The University of Gondar, Gondar, Ethiopia

## Abstract

**Background:**

Tuberculosis is a global emergency predominantly affecting developing countries. HIV has been the single most important reason for acquisition of tuberculosis for many patients. Conversely, tuberculosis can result in rapid progression of HIV disease. Ethiopia is a country affected seriously by HIV and tuberculosis. The main aim of this study is assessment of the types of tuberculosis and the extent of HIV infection among tuberculosis patients visiting private health institutions in Amhara region of Ethiopia.

**Methods:**

The study used a cross sectional method with data collected using well structured pretested questionnaires containing socio-demographic and clinical variables including HIV serostatus. The setting is tuberculosis treatment sites situated at 15 private health institutions in Amhara region.

**Results:**

A total of 1153 TB patients were included. The proportions of smear positive pulmonary TB, smear negative pulmonary TB, isolated extrapulmonary TB and disseminated TB cases were found to be 29.6%, 22.2%, 43.9% and 2.9%, respectively. TB lymphadenitis accounted for about 61% of the extrapulmonary cases followed by TB pleurisy (10.6%). Seventy percent of the patients had undergone HIV test, and 20% of them were HIV positive. Marital status, patient residence and type of TB are the major determinants of co-infection.

**Conclusion:**

The occurrence of pulmonary tuberculosis is relatively low. Tuberculosis/HIV co-infection is also lower than other reports.

## Background

Tuberculosis (TB) is a global emergency posing a significant threat to people of all nations. Despite the advance of DOTS program (Directly Observed Therapy Short Course), TB is still one of the leading causes of death worldwide particularly in the developing countries [1-4]. Failure of TB programs in sub-Saharan African countries is largely due to a parallel HIV epidemic in the continent [5]. Africa accounts for about 70% of the global burden of co-infection with TB and HIV the majority being from sub-Saharan African countries which are frontline people facing challenges from infectious diseases [6,7].

TB and HIV/AIDS are inextricably linked in that one paves the way for the other. As HIV prevalence increases, TB rates have also skyrocketed. Currently about one third of the world’s population are infected with TB bacterium although significant proportion do not show signs and symptoms [8]. The complex relation between the two diseases may affect TB cases finding due to atypical presentation of TB and the presence of co-infections, which may mimic the presence of TB [5]. The risk of developing TB among people infected with HIV/AIDS is ten times higher than those without HIV infection. The primary responsible factor for this is the profound immunodeficiency associated with HIV infection. Although the chance of acquiring TB is progressively increases with the advancement of HIV disease stage, TB tends to occur at any time in the course of HIV disease [6]. It is the leading specific diagnosis among patients living with HIV/AIDS [9].

Treatment of TB in patients with HIV infection is an important step in delaying HIV disease progression. Hence screening patients with a possible diagnosis of TB for HIV infection is a crucial intervention. HIV screening also helps to prepare a special treatment arrangement for those living with HIV/AIDS (like closer follow up and provision of ART for eligible subjects). This also goes in line with providing TB preventive therapy for those patients with HIV infection which helps in reducing the incidence of TB among HIV patients [10,11].

The prevalence of HIV infection among patients in TB clinical settings is high, up to 80% in some countries [12]. The burden of TB is significant in Ethiopia. It is one of the severely affected countries in the world (ranking 7th in the world and 3rd in Africa). The country is also seriously stricken by the HIV epidemic which is extremely dynamic and growing worldwide. Currently the prevalence of HIV is estimated to be 2.1%. According to the World Health Organization the prevalence of TB infection in Ethiopia is 546 per 100,000 people and 41% of them are estimated to have co-infection with HIV [13,14]. A similar prevalence of HIV infection among TB patients has been seen in other African countries as well (Abidjan, Ivory Coast – 40.2%) while still low prevalence has been seen in some others (southern Africa – as low as 1.4%) [15].

To the authors’ knowledge, there is no study done in Ethiopia that shows TB burden and HIV coinfection. In addition the contribution of private health institutions in TB case detection and the overall care is not documented. Studies are lacking, but are important to design how private institutions should contribute.

This study aims at studying the types of TB and the prevalence of HIV infection among TB patients at private-profit healthcare settings in Amhara region.

## Methods

The study is a descriptive cross sectional study which tries to assess types of TB diagnosis and HIV co-infection among TB patients visiting private health institutions in Amhara National Regional State in the year 2008/09. Out of 32 private health institutions in the region that give treatment and care to TB patients, 15 were selected by using lottery method. Study participants were recruited consecutively throughout all the seasons of the data collection year.

The study was done in 15 different private health institutions of the Amhara National Regional State which represents a significant portion of the whole nation. Private health institutions in these zones which offer therapeutic services to TB patients as well as with HIV screening facilities were included in the study.

Data were collected with the help of structured and pretested questionnaires containing the variables age, sex, marital status, place of residence, type of TB and HIV serostatus. Medical doctors who care for patients were assigned as data collectors at each private institution and were supervised by the investigators. HIV serostatus was determined using rapid tests for HIV. And the diagnosis of TB was made based on the conventional means of diagnosis like clinical diagnosis, laboratory evidences including sputum AFB (Acid Fast Bacilli), pathological investigations and culture of specimens.

Analysis was done using SPSS version 15.0. Analysis is handled by statistical summarizing methods like measures of central tendency, measures of dispersion and tables. T test was done to see if there was age difference between female and male TB patients.

The following definitions were used in explaining patient residences: *Urban*: a setting commonly called a city with most of such facilities like hospitals, municipalities, big hotels, night clubs, video shows, universities, colleges, and a relatively higher trade movement with a rough population size of 100,000 to 250,000 people. *Semi-urban*: smaller towns with high schools, bars, post office and moderate trade activities with population of 20,000 to 100,000 people. *Semi-rural*: small sprouting villages with elementary schools, small number of shops and tea rooms with population of up to 20,000 people. *Rural*: remote farmlands with no facilities inhabited solely by farmers. *Mobile* people were those who have not lived more than 6 months in the area they were found at the time of data collection.

*Cold abscess* is a type of abscess that collects in soft tissues anywhere in the body that was diagnosed clinically or laboratory wise to be due to TB.

Before data collection, ethical approval was obtained from the University of Gondar after submitting the proposal to the Research and Publications Office. Official permission was also obtained from each of the private clinics involved in the study. Moreover, verbal informed consent was obtained from patients.

## Results

A total of 1153 TB patients are included in this study. Male patients account for 53.4% of the study subjects. The mean age was 31.6 years (Standard deviation: 13.5; range: 2 to 82). The majority of the study participants are aged between 20 and 40. T-test showed a significant difference in mean age between male and female patients (Mean age = 3.8 years; 95% CI: 2.2 to 5.4).

Marital status was assessed by classifying patients into two groups (those who had never married and those who were married at least once). This was done to see the effect of history of marriage on the prevalence of HIV among TB patients. A total of 1136 patients (98.5%) responded to the question, and 795 (70%) had been married at least once.

A total of 692 (60%) of the study participants lived in rural or semi-rural settings. The rest lived in either urban or semi-urban areas mostly.

Regarding the types of TB, 590 (51.8%) participants were diagnosed with pulmonary TB (PTB); 337( 57%) of whom were smear positive PTB patients. Extrapulmonary TB is seen in 500 (44%) of the patients. Isolated extrapulmonary TB cases were composed of 8 different sites of involvement. The majority, 293 (60.8%), of the cases whose extrapulmonary TB (EPTB) type was specified were TB lymphadenitis (Tables 1 and 2). The type of EPTB was not documented for 18 EPTB patients.

**Table 1:** Types of TB identified at selected private health institutions in Amhara National Regional State, 2008/09

**Types of TB**	**Count (percentage)**
Smear positive PTB	337 (29.6)
Smear negative PTB	253 (22.2)
Isolated extrapulmonary TB	500 (43.9)
Disseminated TB	33 (2.9)
Unspecified pulmonary TB^*^	17 (1.5)
Total	1140 (100)^**^

^*^The smear result of 17 pulmonary TB patients was not documented.

^**^There are 13 patients whose TB type was not documented.

**Table 2:** Types of isolated extrapulmonary TB cases at private health institutions in Amhara National Regional State, 2008/09

**Types of isolated extrapulmonary TB identified**	**Count (percentage)**
TB lymphadenitis	293 (60.8)
TB pleurisy	51 (10.6)
TB peritonitis	45 (9.3)
TB of the spine	34 (7.1)
Cold abscess	25 (5.2)
Skin TB	14 (2.9)
TB arthritis	12 (2.5)
TB osteomyelitis	8 (1.7)
Total	482 (100)^*^

^*^The type of EPTB was not documented for 18 EPTB patients.

TB patients are checked for any possible contact with a chronic cougher at home or in their vicinities. 163 (14.4%) patients reported that they had some sort of contact with a chronic cougher.

HIV tests were offered for all TB patients encountered in the study period. However, 334 (29.3%) patients did not undergo the testing. Out of those patients tested for HIV, 161 (20%) tested positive. The HIV prevalence among different categories of patients was assessed. The results are summarized as in Table 3.

**Table 3:** HIV status of TB patients in relation to their sociodemographic characteristics at selected private health institutions in Amhara National Regional State, 2008/09

**Patient category**	HIV status^*^			**Total, N (%)**
	**Positive, N (%)**	**Negative, N (%)**	**Test not done, N (%)**	
Sex				
Male	73 (12.2)	344 (57.4)	182 (30.4)	599 (53.4)
Female	86 (16.5)	297 (56.9)	139 (26.6)	522 (46.7)
Age Group				
0-14 years	0 (0.0)	36 (58.1)	26 (41.9)	62 (5.5)
15-24 years	21 (7.3)	177 (61.7)	89 (31.0)	287 (25.7)
25-29 years	39 (18.1)	122 (56.5)	55 (25.5)	216 (19.3)
30-59 years	99 (20.0)	271 (54.7)	125 (25.3)	495 (44.3)
60 years and above	1 (1.7)	35 (60.3)	22 (37.9)	58 (5.2)
Marital Status				
Not married at all	38 (11.3)	182 (54.0)	117 (34.7)	337 (30.1)
Married at least once	120 (15.3)	454 (58.0)	209 (26.7)	783 (69.9)
Patient Residence				
Urban	50 (22.5)	88 (39.6)	84 (37.8)	222 (19.7)
Semi urban	59 (27.7)	75 (35.2)	79 (37.1)	213 (18.9)
Rural	18 (3.7)	368 (76.5)	95 (19.8)	481 (42.6)
Semi rural	31 (15.0)	102 (49.3)	74 (35.7)	207 (18.4)
Mobile	1 (20.0)	4 (80.0)	0 (0.0)	5 (0.4)
Type of TB				
Smear positive PTB	52 (15.6)	226 (67.9)	55 (16.5)	333 (29.5)
Smear negative PTB	48 (19.3)	71 (28.5)	130 (52.2)	249 (22.1)
Isolated EPTB	52 (10.5)	326 (65.6)	119 (23.9)	497 (44.1)
Disseminated TB	8 (25.0)	15 (46.9)	9 (28.1)	32 (2.8)
Unspecified PTB	0 (0.0)	0 (0.0)	17 (100)	17 (1.5)

^*^The totals under the categories above vary because the data for each category were not complete. Some patients’ data were not documented, and it was difficult to trace patients after they were sent home.

## Discussion

This study showed that TB occurs in all age groups of the study area: children, adults as well as the elderly. However, adults take the major share. The mean ages of other TB studies are similar to the ones identified in this study [16,17]. A significant age difference was seen among female and male TB patients.

Possible factors which may be related to TB HIV co-infection like marital status, patient residence and type of TB were investigated in this study. Majority of the patients were married at least once. A number of patients had marital relations even more than once. Regarding patient residence, though the private health institutions studied in this research are mainly located in urban settings, most of the TB patients encountered were from rural or semi-urban settings. This is a reflection of the fact that TB is mainly a disease of the poor and rural people. Concerning HIV, co-infection occurred more in urban and semi-urban settings (Table 3). This shows HIV is mainly a disease occurring in more numbers in urban areas. This is in line with data from other observations [14,18].

The proportion of PTB patients in this study is low when compared to a similar study done in Gondar town which showed PTB prevalence of 78.7% [19]. In almost half of the cases the current study showed the diagnosis of isolated EPTB case which is much higher than expected. However, the commonest case of isolated EPTB was TB lymphadenitis. It accounted for about 61% of the extrapulmonary cases, followed by TB pleurisy (10.6%). Diagnostic issues might be considered as partial explanation for the higher percentage of TB lymphadenitis. As most studies suggest, smear negative PTB and EPTB are commonly encountered in HIV co-infected patients. This study showed HIV prevalence of 20%. This percentage was actually brought about by considering only the voluntarily tested individuals. Quite a big number of patients were not screened for one or another reason [14,20-23].

A study done by Datiko DG et al. showed similar HIV prevalence among TB patients, the rate of HIV co-infection being higher in patients from urban areas [24]. A similar research done in Oromia region showed a co-infection rate of 21% which is comparable to this study [25]. However higher percentages were found in many other studies done in different parts of the country. Some of these researches showed a prevalence of co-infection as high as even more than half of the cases. The rate of TB and HIV co-infection in Ethiopia was shown by other studies to be in the range of 40-70%. Extending the situation to outside Ethiopia, in parts of sub-Saharan Africa up to 70% of TB patients are co-infected with HIV [26]. This link between HIV and TB is not that surprising considering the pathogenetic links between the two diseases [19,27-29]. The differential occurrence of co-infection in rural and urban areas can be clearly explained because of urban dominance in the occurrence of the HIV epidemic.

Unmarried TB patients and urban dwellers were found to have higher proportions of HIV positive results, whereas there was no marked serostatus difference between male and female patients. Unmarried TB patients are likely to have multiple sexual partners and hence are at increased risk of acquiring HIV infection [18].

This study is strong in that it considered many private healthcare institutions that are alternative healthcare areas to government institutions, and hence the results will complement data from government institutions. However, the fact that different healthcare professionals were involved in assessing patients and making diagnoses might have resulted in information bias. Seasonal selection bias may not be a serious issue as patients who came at different seasons of the year were recruited to the study.

## Conclusion

In conclusion, most TB patients in this study are from rural areas. The percentage of PTB among the study participants was found to be low compared to other studies in the country. Higher proportion of EPTB, particularly TB lymphadenitis, was seen. The prevalence of TB HIV co-infection was lower than that seen in different studies across the country.

Considering the predominance of TB lymphadenitis and smear negative PTB, diagnostic criteria used might need serious scrutiny, as most of the private institutions lack some of the advanced tests like culture and sensitivity and pathologic examinations. Appropriate integrations of TB and HIV interventions have to be given more emphasis particularly in urbanized settings as these were found to have a relatively higher co-infection.

## Competing interests

The authors declare that they have no competing interests.

## Authors’ contributions

FG was involved in the data collection and analysis. GAA did the data entry, data analysis and write up of the article. Both authors read and approved the final manuscript.

## Pre-publication history

The pre-publication history for this paper can be accessed here:

http://www.biomedcentral.com/1471-2458/14/319/prepub
